# Whole genome-wide chromosome fusion and new gene birth in the *Monopterus albus* genome

**DOI:** 10.1186/s13578-020-00432-0

**Published:** 2020-05-20

**Authors:** Yibin Cheng, Dantong Shang, Majing Luo, Chunhua Huang, Fengling Lai, Xin Wang, Xu Xu, Ruhong Ying, Lingling Wang, Yu Zhao, Li Zhang, Manyuan Long, Hanhua Cheng, Rongjia Zhou

**Affiliations:** 1 Hubei Key Laboratory of Cell Homeostasis, College of Life Sciences, Wuhan University, Wuhan, 430072 People’s Republic of China; 2 Department of Ecology and Evolution, University of Chicago, Chicago, 60637 USA

**Keywords:** Genomics, Evolution, Chromosome, Vertebrates

## Abstract

**Background:**

Teleost fishes account for over half of extant vertebrate species. A core question in biology is how genomic changes drive phenotypic diversity that relates to the origin of teleost fishes.

**Results:**

Here, we used comparative genomic analyses with chromosome assemblies of diverse lineages of vertebrates and reconstructed an ancestral vertebrate genome, which revealed phylogenomic trajectories in vertebrates. We found that the whole-genome-wide chromosome fission/fusions took place in the *Monopterus albus* lineage after the 3-round whole-genome duplication. Four times of genomic fission/fusions events resulted in the whole genome-wide chromosome fusions in the genomic history of the lineage. In addition, abundant recently evolved new genes for reproduction emerged in the *Monopterus albus* after separated from medaka. Notably, we described evolutionary trajectories of conserved blocks related to sex determination genes in teleosts.

**Conclusions:**

These data pave the way for a better understanding of genomic evolution in extant teleosts.

## Introduction

Fish is an enormously species-rich group in vertebrates. Total number of fish species is over 35,200, and approximately 2000 new fish species have been named in the past 5 years [1]. On the earth, fishes account for more than half of extant vertebrate species, and show a rich evolutionary diversity [2]. Compared to land vertebrates, fishes display remarkable variations in morphological and physiological adaptations, which is one of successful group of vertebrates evolutionarily. Fishery is an important part in the economy of many nations. Fish not only provides food for people, but also has immense values to humans, such as in recreations and sports. With in-deep study, some have also become model species in biology, ecology, medicine, and fishery, such as zebrafish and medaka [3, 4].

Although belonging to different orders, zebrafish (Cypriniformes) and medaka (Beloniformes) show their respective advantages as vertebrate model organisms for biology research. Several other fish species have distinct features in evolution. For example, pufferfish *Takifugu rubripes* (Tetraodontiformes) is a model organism owing to a particularly small and compact genome of 400 Mb [5], and stickleback fish (Perciformes) has been widely used to study adaptive evolution, because of its plasticity to new niche by adaptive radiation [6]. The teleost *Monopterus albus* is a new model species for evolution, genetics and development [2]. The *Monopterus albus* has an unusual reproductive strategy, known as protogynous hermaphroditism; it begins life as a female, but transform to a male through an intersex stage naturally. This reproductive advantage ensures successful establishment of new colonies when isolated small populations appear with extremely biased sex ratios during natural selection. Natural sex reversal in *Monopterus albus* was first reported in 1944 by Liu [7]. Three years later, Bullough discussed it in Nature [8]. Aerial respiration was a crucial step in the origin of tetrapods. As an air-breather, *Monopterus albus* is an ideal species for deepening our understanding of vertebrate evolution from survival in the sea to survival on land. *Monopterus albus* can breathe air, and it is capable of surviving for a long period without water. Its physiological features, including amphibious ability, make the species a successful invader around the globe. It is distributed mainly in China, Japan, Korea, Thailand, Lao, Indonesia, Malaysia, Philippines, India and Australia and United States [2]. In fact, it has the potential for disrupting currently threatened ecosystems [9]. Furthermore, *Monopterus albus* has the smallest haploid number (2*n *= 24) of chromosomes among those of most freshwater fishes (2n = 24–446) and the fewest chromosome arms (all telocentric) with no heteromorphic sex chromosome [10], which is an ideal material for chromosomal evolution studies.

Rapid establishment of sex determination after whole-genome duplication (WGD) is essential for survival of species. Understanding how and why the two sexes arise has been a topic of great interest since Darwin’s time, garnering both theoretical and observational efforts [11, 12]. The sex chromosomes XX/XY in mammals and ZZ/ZW in birds evidently evolved from different pairs of autosomes independently within the last 360 million years [13–16]. The W chromosome evolved in parallel with the Y chromosome, preserving ancestral genes through purifying selection [17]. Recombination suppression on the chromosomes X/Y and Z/W has resulted in degradation and differentiated sex chromosomes [18–20]. For example, the human Y chromosome has retained a few dozen genes with male-specific functions. Notably, several mammals have completely lost their Y chromosome, resulting in XO sex determination systems [21, 22]. The endpoint and fate of Y degradation have sparked intense interest in recent years [20, 23]. These ancient sex chromosomes can provide information about the evolutionary fates of sex chromosomes, but they shed little light on the early stages of sex chromosome evolution in vertebrates [24]. In many vertebrate species, the sex chromosomes are morphologically undifferentiated and remain largely identical [25, 26]. For example, a single missense single nucleotide polymorphism in *amhr2* on XY, which emerged at approximately 40 MYA, can determine sex in pufferfish (*Takifugu rubripes*) [27]. However, the chromosome evolutionary mechanisms underlying sex determination remain poorly understood, particularly in teleosts, which represent half of all living vertebrate species.

Recently, we sequenced and assembled the whole genome of the teleost *Monopterus albus* at chromosome level [28]. Given the smallest chromosome number among teleost fishes, it is interesting to see how third whole-genome duplication (the 3R WGD) occurred in the *Monopterus albus* lineage, because 3R WGD occurred in the other teleost lineages after divergence from the Holostei [4, 29–32]. Taking advantage of comparative genomics and the unique genome structure of *Monopterus albus*, we described the phylogenomic events and evolutionary history of *Monopterus albus*, focusing on the genomic scenario behind the 3R WGD. By reconstructing an ancestral vertebrate genome using available chromosomal assemblies of related vertebrates along with the assembly from the *Monopterus albus* chromosomes, we systematically analyzed genomic events from 2R to 3R to post-WGD, and provided genomic history of teleost fishes based on striking genomic features. These analyses revealed the whole-genome-wide chromosome fission/fusion events and abundant recently evolved new genes for testis development in the *Monopterus albus* lineage after separated from medaka ~ 70 MYA. In addition, we described evolutionary trajectories of conserved blocks related to sex determination genes in teleosts and three independent origins of corresponding loci/chromosomes in vertebrates.

## Materials and methods

### Analysis of gene duplication in the *Monopterus albus* genome

Duplicated genes in *Monopterus albus* were analyzed by alignment of the protein sequences within the genome by Blastp (*E* value < 10^−7^). The *Monopterus albus* whole-genome data were uploaded from GenBank under the accession AONE00000000 [28]. Protein sequence pairs with identity and coverage of over 60% were selected as candidate gene pairs. To confirm real gene duplications, the paired sequences were blasted against the NCBI non-redundant protein database to confirm that they were the same genes. The filtered gene pairs were mapped onto the genome with the program Circos (version 0.69) [33].

### Ancestral genome reconstruction and chromosome evolution

Models of vertebrate genome evolution were deduced from the phylogenetic tree [31, 34]. Datasets of gene annotations and protein sequences of each species were obtained from Ensembl (release 95), including those of medaka, *Tetraodon*, stickleback, zebrafish, tongue sole, spotted gar, *Xenopus*, lizard, snake, chicken and human. All the protein sequences were aligned to the protein databases of *Monopterus albus* and other genomes using Blastp with *E* value < 10^−7^, and lower quality datasets (identity < 30%) were filtered. The filtered genes were mapped onto the genome with the program Circos (version 0.69). MCScanX [35] was used to find conserved syntenic blocks (≥ 5 genes, *E* value < 10^−5^) between species. Insertions and deletions of other genes (1/25, 1 insertion/deletion in a block of 25 genes) were allowed within the blocks, based on adequate information for comparison and block conservation among species. Chromosome synteny analysis using the data from Ensembl was performed using the JCVI package (10.5281/zenodo.31631), with a parameter “–minspan = 5”. A chromosome model of the vertebrate ancestor was constructed with the conserved syntenic blocks shared by all genomes. The numbers of genes in the blocks between *Monopterus albus* and pre-3R WGD species were 3983 (chicken), 2448 (human) and 5680 (spotted gar), which were approximately half those of the 3R WGD fishes (8256 in zebrafish, 7791 in *Tetraodon*, 9575 in medaka and 10,297 in stickleback). The final ancestral genome model was inferred from all the species genomes by the maximum parsimony method based on both duplicated genes and conserved syntenic blocks. The evolutionary relationship of the chromosomal blocks from the ancestral genome to the different species genomes was analyzed and displayed by a Perl script.

### *Ka* and *Ks* value calculations

Nonsynonymous substitution (*K*a) and synonymous substitution (*K*s) values were calculated based on alignments of the coding regions of paired genes between two species. CDS sequences without untranslated regions of two genes were extracted from the Ensembl and the *Monopterus albus* CDS databases and then aligned by ClustalX 2.0 [36] with the default parameters. The alignments were sent to MEGA 6, and *K*a and *K*s values were calculated using the Nei-Gojobori method with Kimura’s two-parameter model [37].

### Gene coverage and expression levels

Gene expression levels were calculated by the ‘reads per kb per million reads’ (RPKM) method [38] to eliminate the influence of sequencing discrepancies and differences in gene length. Therefore, the gene expression levels were directly comparable among different tissue samples. When a gene had more than one transcript, the longest transcript was used to calculate its coverage and expression level. Total transcriptome reads, which were transformed into expression levels, were mapped onto the contig assembly using the program TopHat [39] (version 1.3.3). The *Monopterus albus* transcriptome data were uploaded from Gene Expression Omnibus GSE43649 [28].

### Analysis of new genes

The pipeline for the identification of new genes was divided into two parts (Additional file 1: Fig. S1), based on the methods described previously [40]. First, we used 20,456 *Monopterus albus* protein sequences (predicted by FGENESH and GENSCAN) [28] to search against vertebrate protein sequences (1,562,657) and invertebrate protein sequences (3,715,843) by Blastp, and 4,221 genes were identified that did not have any homologous protein sequences in any organism. After we excluding the genes that were either too short (180 bp) or lacked start and stop codons, 2888 genes were retained as candidate orphan genes in *Monopterus albus* based on definition of no homologues in closely related lineages. RNA-seq datasets were used to confirm that 1533 genes were new protein-coding orphan genes. Second, 24,056 protein sequences in *Monopterus albus* are self-searched by Blastp, and candidate pairs were identified. These genes are compared with coding genes of the related fish species (Tilapia, Medaka, Stickleback, *Tetraodon* and Zebrafish), and then those genes with no homologue among these species were defined as duplication new genes. By analysis of differences in exon number between parental genes (multiple exons) and candidate new genes (1 exon), the corresponding genes with one exon were defined as new genes arising from retroposition. Relative expression levels of the new genes of *Monopterus albus* were calculated using log_2_(RPKM +1). The statistical hypothesis was tested using the Mann–Whitney *U* test and Kruskal–Wallis test in the R language. The chromosome distributions of new genes, orphan genes and other genes were statistically tested by χ^2^ tests.

## Results

### Evolutionary trajectory of the chromosomes in fishes

**Fig. 1:**
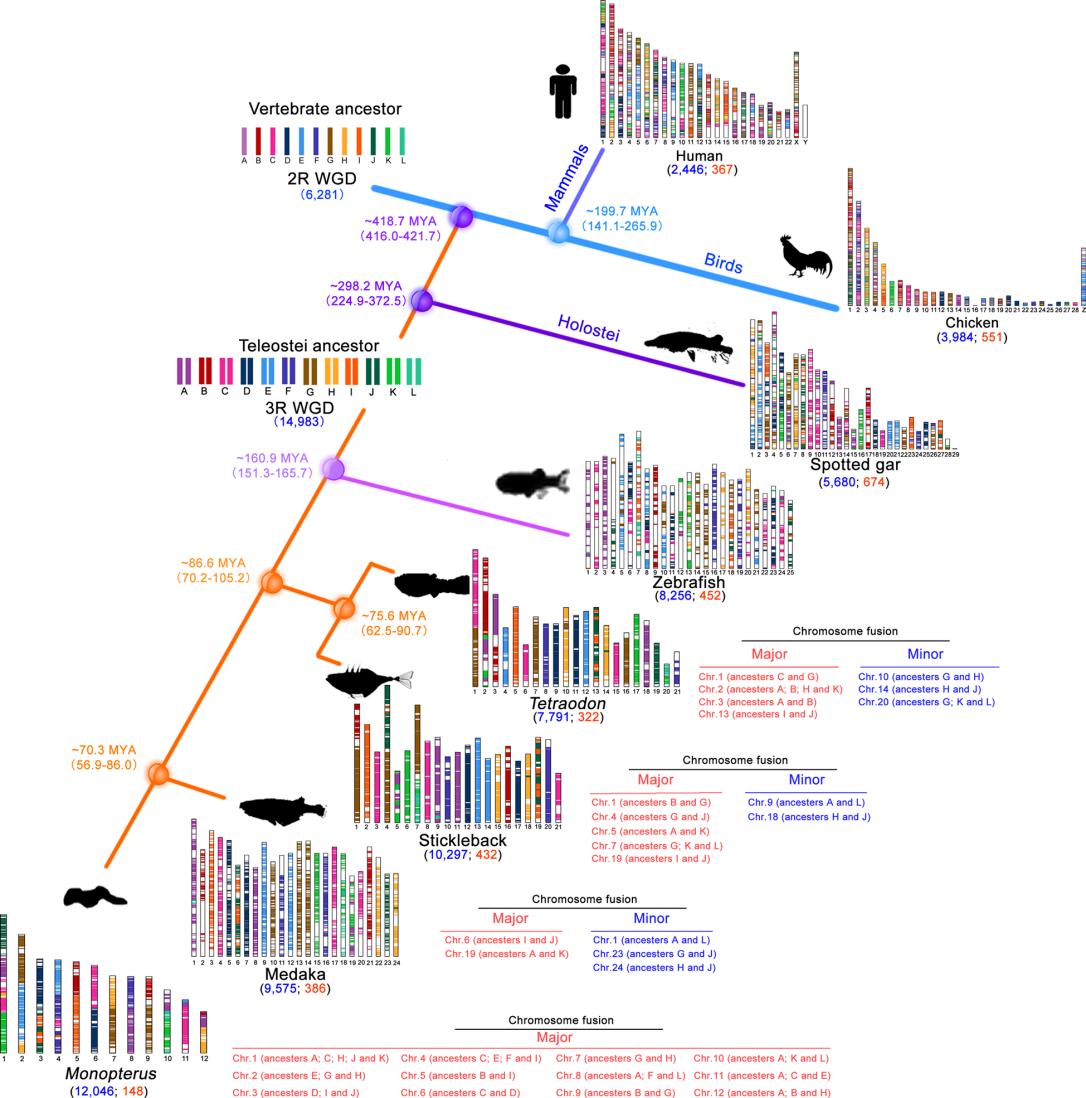
Phylogenomic trajectories of extant teleost fishes at chromosomal levels and chromosomal fusion events. Vertebrate genomes evolved from 12 ancestral chromosomes through chromosomal loss, fission and fusion, and syntenic block recombination, in addition to whole-genome duplications. The figure depicts the distribution of conserved syntenic blocks of chromosomes in teleosts (*Monopterus albus*, medaka, stickleback, *Tetraodon* and zebrafish), Holostei (spotted gar), birds (chicken) and mammals (human). Genomic blocks in each species originating from the ancestral chromosomes are shown in the same color. An additional genome duplication (the 3R WGD) took place in the teleost lineage after divergence from the Holostei (spotted gar), approximately 298 MYA, while the second round of genome duplication (the 2R WGD) occurred in the common ancestor of mammals, birds, Holostei and teleosts, approximately 500 MYA. Major and minor events of chromosome fusion in each species are shown on the right panels. Average divergence times are shown on the branch nodes. The number of conserved genes is shown in blue, and the number of conserved blocks is in orange

To investigate the evolutionary trajectory of the chromosomes in fishes with addition of the *Monopterus albus* chromosome data based on phylogenetic relationship, we first confirmed 3R WGD occurred in the *Monopterus albus* lineage. Circos mapping showed that many duplicated genes existed in pairs among chromosomes (Additional file 1: Fig. S2), suggesting that the 3R WGD had occurred in the *Monopterus albus* genome as the other teleost fishes did. Comparative mapping of conserved syntenic blocks (CSB) among diverse vertebrate genomes was used to trace the origin and evolution of the chromosomes (Additional file 1: Fig. S3). The evolutionary distribution of the CSBs among teleosts (*Monopterus albus*, medaka, stickleback, *Tetraodon* and zebrafish), Holostei (spotted gar), birds (chicken) and mammals (human) was determined according to phylogenetic tree reconstruction, and we identified the evolutionary relationship of these diverse vertebrate genomes based on the 12-chromosome model using a common-ancestor gene set of the ancestral vertebrate genome (proto-chromosome A to L) [31, 34]. Among the 2R species without the 3R WGD, the tetrapod (chicken and human) and Holostei (spotted gar) genomes have undergone extensive chromosomal fission and block recombination from the ancestral vertebrate genome (Fig. 1). Furthermore, these 2R vertebrates retained smaller CSBs (≤ 14 genes/block) than 3R teleosts (Fig. 1; Additional file 1: Fig. S4). The 3R WGD took place in the teleost lineage after divergence from the Holostei, approximately 298.2 MYA, and caused the number of chromosomes to double in the teleost ancestor. After the 3R WGD, the teleosts underwent broadly genomic events, including extensive gene/region loss, block recombination and chromosomal fission/fusion (Fig. 1). For example, the chromosomal number in *Monopterus albus* decreased from n = 24 to n = 12. In addition, zebrafish diverged from other fishes ~ 160 MYA and had a relatively small CSB ranging from 10 to 26 genes/block, whereas the other teleosts with a short history (< 90 MYA) have retained a large CSB with > 65 genes/block (Additional file 1: Fig. S4), indicating that early speciation accumulated genomic variations during evolution, while these CSBs have homoplasic characters in the lately diverged fish clades in particular.

### From partial to whole genome-wide chromosome fusion in fishes

**Fig. 2:**
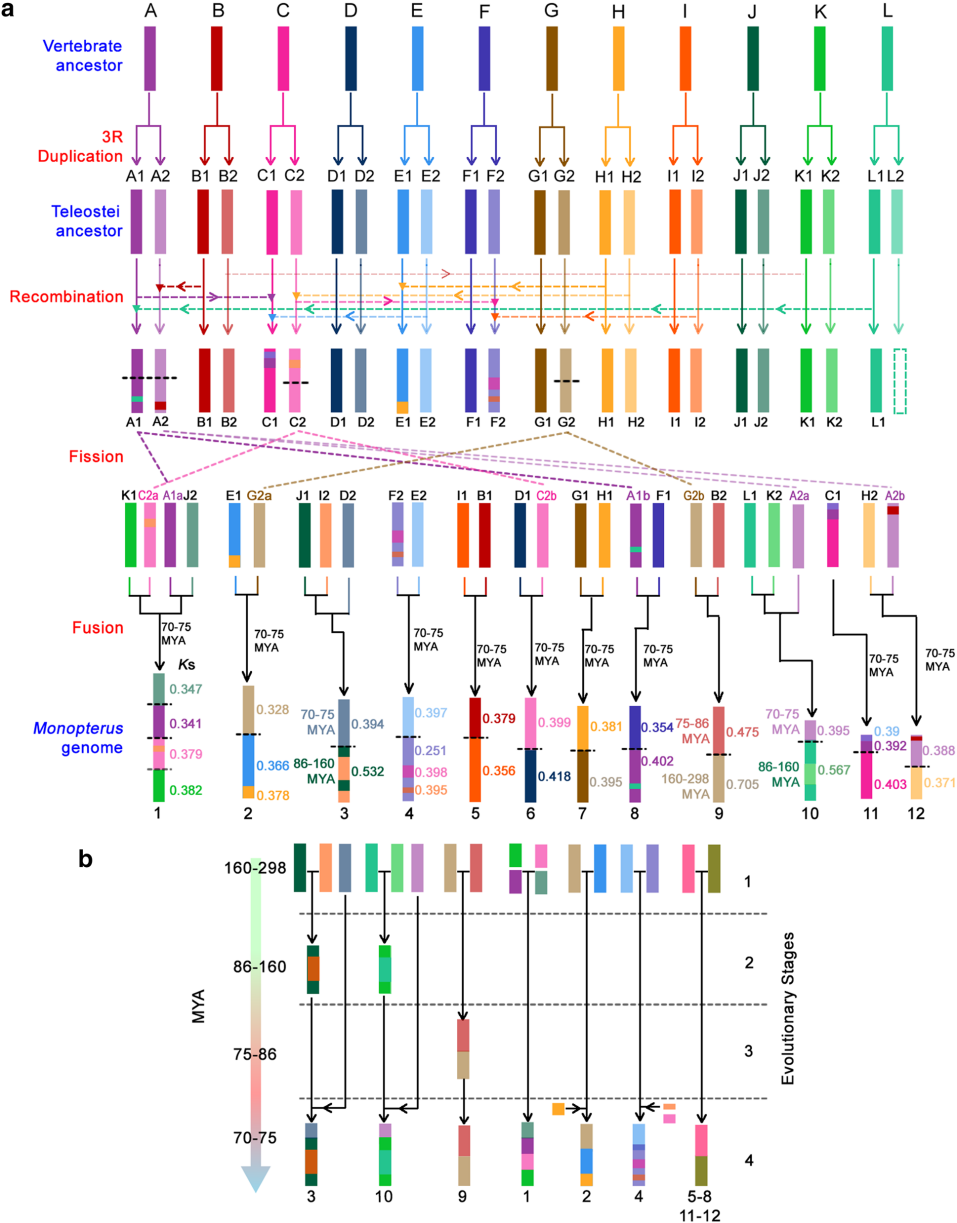
Whole-genome-wide chromosome fission/fusions in the *Monopterus albus* lineage. **a** The diagram depicts the formation of the 12 chromosomes of *Monopterus albus* from 12 vertebrate ancestor chromosomes (A to L). After the 3R WGD, 24 ancestral chromosomes (A1, A2, B1, B2,… L1, L2) underwent block recombination (A1 to C1, B1 to A2, L1 to A1, E2 to C1, H2 to C2, C2 to F2, H1 to E1, and I2 to F2), chromosome loss (e.g., L2), fission (e.g., A1, A2, C2 and G2) and fusion events. Finally, 12 chromosomes formed in *Monopterus albus*. *K*s values are calculated based on CDS alignments between *Monopterus albus* and its close relatives at each evolutionary time. These values are used to gauge the evolutionary ages (MYA) of the chromosome fusion events and block recombination. **b** Four evolutionary stages in the *Monopterus albus* genome. For chromosomes 3 and 10, two rounds of fusion events occurred approximately 86-160 MYA and 70–75 MYA, respectively. One fusion event took place approximately 75–86 MYA on chromosome 9, and another occurred 70–75 MYA and involved all other chromosomes. Block recombination also occurred on chromosomes 2 and 4, approximately 70–75 MYA. These events generated four evolutionary stages in the *Monopterus albus* genome

Chromosome number has halved during the evolution of *Monopterus albus*, which is attributable to either chromosome loss or fusion. Comparative mapping of the CSBs clearly indicated that chromosomal fission/fusions mainly occurred in the fish lineages after separated from zebrafish (Fig. 1). Major events of fission/fusions were involved in large chromosomal regions, even in whole chromosomes from ancestors, while minor events took place, often in fusion of small chromosomal fragments. Notably, the *Monopterus albus* genome, compared to those of its relatives, underwent whole-genome-wide chromosomal fission/fusions (WGCF), in addition to rearrangement events involving syntenic blocks, after diverging from medaka ~ 70 MYA (Figs. 1, 2a).

After the 3R WGD, 24 ancestral chromosomes in Teleostei (A1, A2, B1, B2,… L1, L2) underwent block recombination, chromosomal loss (e.g., L2), fission (e.g., A1, A2, C2 and G2) and fusion events (Fig. 2a). For example, a *Monopterus albus* -specific fusion event occurred at approximately 70-75 MYA to form chromosomes 2 and 4. Three major block recombination events (C2 to F2, H1 to E1, and I2 to F2) also occurred on the chromosomes 2 and 4 (Fig. 2a**)**. To confirm the chronological order of these events, we used the *K*s value (synonymous substitutions per synonymous site) to measure evolutionary time. The calculated *K*s values based on CDS alignments between *Monopterus albus* and close relatives are consistent with the evolutionary times of species divergence. Thus, these cross-species comparisons indicated four evolutionary stages of the formation in the *Monopterus albus* genome (Fig. 2b). Stage 1, the first in chronological order, involved the formation of 24 chromosomes through the 3R WGD, approximately 160–298 MYA; stage 2 included the first round of chromosomal fusion events on chromosomes 3 and 10, approximately 86–160 MYA; stage 3 was a chromosomal fusion event on chromosome 9, approximately 75–86 MYA; and stage 4, the most recent, which occurred ~ 70–75 MYA, included the formation of all other chromosomes, e.g. chromosomes 2 and 4, through chromosomal fusion and block recombination events. Thus, after the 3R WGD approximately 300 MYA to the genome ~ 70 MYA, the genomic history of *Monopterus albus* through WGCF, genomic reorganization and diploidization spanned approximately 230 million years.

### Evolutionary trajectories of conserved blocks related to sex determination genes

**Fig. 3:**
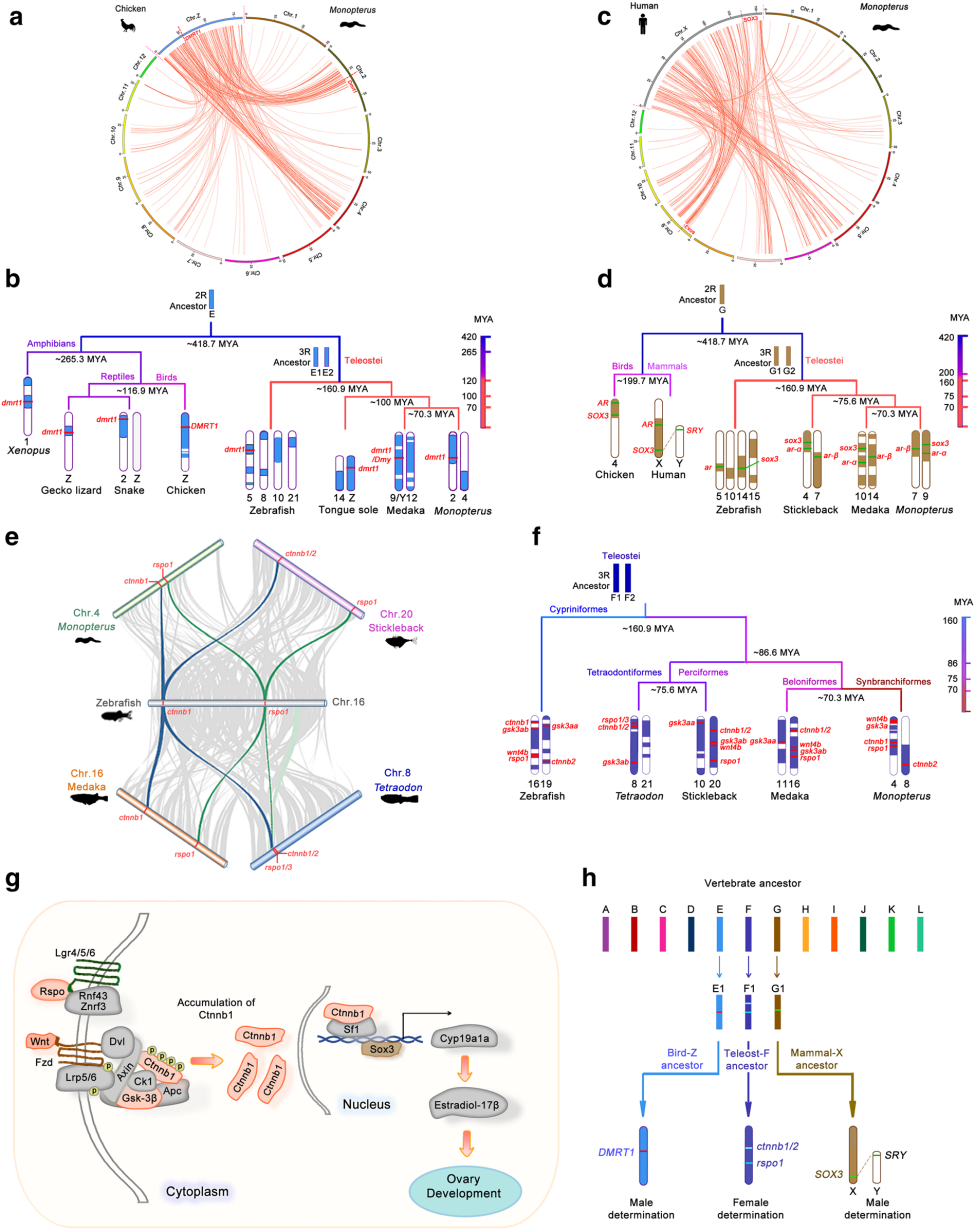
Evolution of chromosome Fs and multiple independent origins of conserved blocks related to sex-determining genes. **a** Circos shows the syntenic relationships of the ohnologous genes between the chicken Z and *Monopterus albus* chromosomes. Red inner links show the ohnologous genes between the chromosomes in *Monopterus albus* corresponding to the chicken Z chromosome. The male-determining gene *dmrt1* is highlighted in red. **b** The schematic diagram depicts the evolutionary trajectory of the syntenic blocks of the bird-like Z chromosome in birds (chicken), reptiles (gecko lizard and snake), amphibians (*Xenopus*) and teleost fishes from the ancestral chromosome E in 2R approximately 418.7 MYA. Syntenic blocks shared with the bird-like Z chromosome are shown in blue. The male-determining gene *dmrt1* is highlighted in red. An estimated divergence time ruler in MYA is shown on the right. **c** Syntenic relationships of the ohnologous genes between the human X and *Monopterus albus* chromosomes. Red inner links show the ohnologs between the chromosomes in *Monopterus albus* corresponding to the human X chromosome. *SOX3*, an ancestor gene of the approximately sex-determining gene *SRY,* is highlighted in red. **d** Evolutionary trajectory of the syntenic blocks of the human X chromosome in chicken and teleost fishes, from the ancestor chromosome G in 2R approximately 418.7 MYA. Syntenic blocks shared with the X chromosome are shown in brown. The male-determining gene *SRY* on chromosome Y approximately evolved from *SOX3* on chromosome X in mammals. The chromosomal regions of representative markers *SOX3* and *AR* are conserved across species. An estimated divergence time ruler in MYA is shown on the right. **e** Conserved synteny among chromosome 16 in zebrafish, chromosome 8 in *Tetraodon*, chromosome 20 in stickleback, chromosome 16 in medaka and chromosome 4 in *Monopterus albus*, analyzed by the JCVI software package. Syntenic blocks of ≥ 5 genes shared among the species are shown in links. Blue links indicate blocks with *β*-*catenin* (*ctnnb1*); green links indicate blocks with *rspo1*. *Tetraodon* has only one *rspo* gene in its genome, homologous to zebrafish *rspo1* and *3*. Chromosome datasets of zebrafish, *Tetraodon*, stickleback and medaka were obtained from Ensembl (release 95). **f** Evolution of the chromosome Fs in the teleost lineage. The ancestor chromosome F in 2R generated a pair of chromosomes, F1 and F2, when the 3R WGD occurred approximately 300 MYA. The chromosome Fs are conserved in teleosts, which include chromosome 16 in zebrafish, chromosome 8 in *Tetraodon*, chromosome 20 in stickleback, chromosome 16 in medaka and chromosome 4 in *Monopterus albus*. Key genes in the female determination pathway (*rspo1*, *ctnnb1, wnt4b* and *gsk3a*) conserved on the chromosome Fs across species are highlighted in red. In *Tetraodon, wnt4b* is on a scaffold and has not yet been mapped onto chromosomes. An estimated divergence time ruler in MYA is shown on the right. **g** The Rspo1/Wnt/Ctnnb1 signaling pathway for female sex determination in teleosts. Key factors in the pathway from the chromosome Fs are highlighted in red. In the model, Rspo1 binds cell surface receptors Znrf3/Rnf43 and LGR4/5/6 and inhibits the ubiquitin E3 ligase activities of Znrf3/Rnf43, which leads to stacking of Fzd receptors on the membrane. The accumulation of Fzd can recruit enough of the Wnt ligands and trigger the Wnt signaling response. The signal is then relayed to the downstream complex of phosphorylation and ubiquitination consisting of Gsk3b/Ck1/Dvl/Axin1/Apc in the cytoplasm. Gsk3b and Ck1 phosphorylate Ctnnb1, which is captured by the degradation complex for ubiquitination. Within the large complex, modified Ctnnb1 saturates and inactivates the degradation complex, allowing newly synthesized, unmodified Ctnnb1 to accumulate in the cytoplasm. The Ctnnb1 is then translocated into the nucleus to activate transcription of downstream Wnt target genes, such as *cyp19a1a*. Ctnnb1 as a cofactor, in association with transcription factor Sf1, activates transcription of *cyp19a1a*. Sox3 can also bind to the promoter of *cyp19a1a* and up-regulate *cyp19a1a* expression. Thus, both Ctnnb1 and Sox3 promote the Cyp19a1a-17β-E2 pathway for ovary development. **h** Multiple independent origins of blocks related to sex determination genes. The bird-like Z chromosome originated from ancestor chromosome E. The syntenic blocks with male-determining gene *DMRT1* on it are conserved in vertebrates. The mammalian X/Y chromosomes originated from ancestor chromosome G. The ancestor chromosome F generated the chromosome Fs with female-determining genes *ctnnb1/2* and *rspo1* in the teleost lineage

Comparative mapping of the CSB and chromosome evolution facilitates to trace formation trajectory of sex-associated blocks/chromosomes, and gets insights into sex determination in teleosts in particular. *DMRT1* on the Z chromosome is a male-determining gene in chicken [41]. To trace evolutionary trajectory of the bird-Z chromosome, comparative mapping of the CSB was used among genomes in far-distant vertebrates. Genomic analysis indicated that the syntenic Z blocks were shared among diverse vertebrates from birds, reptiles, amphibians to teleost fishes, which is consistent with the conserved *dmrt1* on their corresponding Z-associated chromosomes (Fig. 3a, b). Notably, these Z-associated chromosomes were originated from common ancestor chromosome E (Figs. 1, 3b). After 3R WGD, the chromosome E duplicated and diploidized into 2 chromosomes in many teleosts, whereas it has evolved and fragmented in zebrafish genome. In particular, the duplicated E chromosomes fused recently with two other chromosomes (G and F) to form chromosomes 2 and 4 around ~ 70.3 MYA, which carry the highest number of chicken Z genes (227 and 183) in the *Monopterus albus* genome (Additional file 1: Figs. S5, S6; Table S1). Both birds and the fish Tongue sole have ZZ/ZW sex determination system with the *DMRT1* on its Z chromosome [42]. Male-determining gene *dmy* in Medaka was duplicated from *dmrt1* during evolution [43, 44]. However, the other vertebrates retained the chromosomal region with *dmrt1* on their autosomes, including chromosome 2 in snake, chromosome 1 in *Xenopus,* chromosome 5 in zebrafish, and chromosome 2 in *Monopterus albus*. These results suggested that the conserved syntenic block with *dmrt1* retained male-determination from ancestor chromosome after 2R WGD, regardless of the Z or autosomes the block located on approximately ~ 500 MYA.

In mammals, male-determining gene is *SRY* on the Y chromosome, which evolved from *SOX3* on the chromosome X [45]. Evolutionary trajectory of chromosome X indicated that the X was originated from ancestor chromosome G, and the corresponding CSBs were conserved on chromosome X in mammals, but on autosomes in chicken (chromosome 4), zebrafish (chromosomes 5, 10, 14 and 15), stickleback (chromosomes 4 and 7), medaka (chromosomes 10 and 14), and *Monopterus albus* (chromosomes 7 and 9). Moreover, the corresponding chromosomes in non-mammals retained *SOX3*, not *SRY* (Fig. 3c, d).

A great challenge is to search female-determining chromosomes/genes in teleosts in particular. By means of the comparative mapping of conserved blocks, we found that 3R WGD generated a pair of common ancestor chromosome F in the teleost lineage ~ 300 MYA. The ancestor chromosome F is evolutionarily conserved in teleosts, and has evolved into chromosomes 16 in zebrafish, 8 in *Tetraodon*, 20 in stickleback, 16 in medaka, and 4 in *Monopterus albus* with *Rspo1*, a key factor involved in ovary development (Fig. 3e, f). To investigate functions of the descendant chromosomes of the ancestor chromosome F, we analyzed Gene ontology (GO) of the genes shared among these chromosomes in zebrafish, medaka, stickleback, *Tetraodon* and *Monopterus albus,* and observed most of these shared genes are enriched in regulation of cell adhesion, tissue morphogenesis and Wnt signaling pathway in biological processes, and nucleoside binding and protein phosphorylation in molecular function (Additional file 1: Fig. S7). Interestingly, the key factors of the Rspo1/Wnt signaling pathway, including Rspo1, Wnt4b, Ctnnb1 and Gsk3a/b, were associated with ovary development (Fig. 3g), which is consistent with functional data [46, 47]. This finding suggests that conserved blocks from the common chromosome F retained genes for female sex determination in the linages of teleosts. Thus, for uniformity, we call these descendants from the common ancestor chromosome F as the Fs (female sex-determinant F) in extant teleosts.

### Newly evolved genes in the *Monopterus albus*

**Fig. 4:**
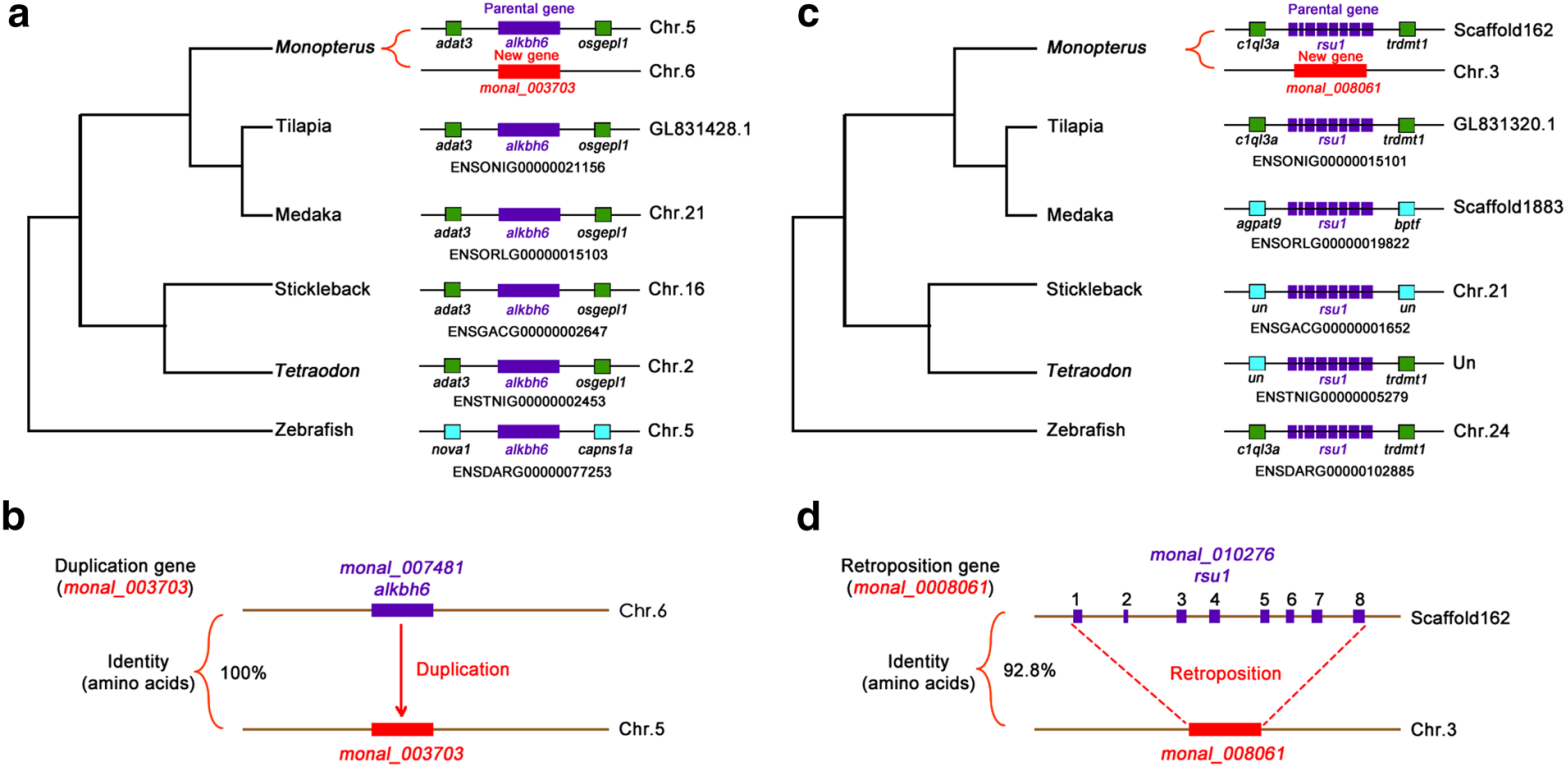
Birth of new genes in the *Monopterus albus* lineage. **a** New genes of duplication type. Syntenic alignments in teleost genomes show that *monal_003703* emerged by duplication from parental gene only in the *Monopterus albus* lineage. **b** New gene (*monal_003703*, red) on chromosome 5 is a duplicated copy of the known parental gene *alkbh6* on chromosome 6 (*monal_007481*, purple). They shared 100% of amino acid identity. **c** Retroposition gene *monal_008061*. Syntenic alignments in teleost genomes show that *monal_008061* emerged only in the *Monopterus albus* lineage. **d** Retroposition gene *monal_008061* is an RNA-based duplicate. A transcribed RNA is reverse transcribed from the gene *rus1* (*monal_010276*) and retroposed into a new position in the genome. Multiple exons (8 exons) was integrated into one exon in the new locus. They shared 92.8% of amino acid identity

Because the sex determination mechanism in *Monopterus albus* evolved after its divergence from related species, e.g., medaka and stickleback, we investigated recently evolved new genes that became fixed in *Monopterus albus* less than 70 MYA, to detect evolutionary innovation that may illuminate how *Monopterus albus* evolved. In total, we identified 94 new genes from recent gene duplications, 2 new genes from retroposition at a threshold of 40%, and 1533 orphan genes (Additional file 1: Figs. S1, S8, Table S2). The generation mechanisms of DNA-based duplications, RNA-based duplications and orphan genes are quite different. The new gene *monal_003703* on chromosome 5 was generated by DNA-based duplication from its parental gene *alkbh6* on chromosome 6 (Fig. 4a, b). Both genes shared 100% of amino acid identity. RNA-based duplication produced the new gene *monal_008061* with one exon by retroposition from its parental gene *rsu1* with eight exons (Fig. 4c, d), and they shared 92.8% of amino acid identity. Syntenic alignments showed that these new genes emerged only in the *Monopterus albus* lineage. Orphan genes have no homologous sequences in the closely related species medaka, tilapia, stickleback, *Tetraodon* and zebrafish (Additional file 1: Fig. S9).

**Fig. 5:**
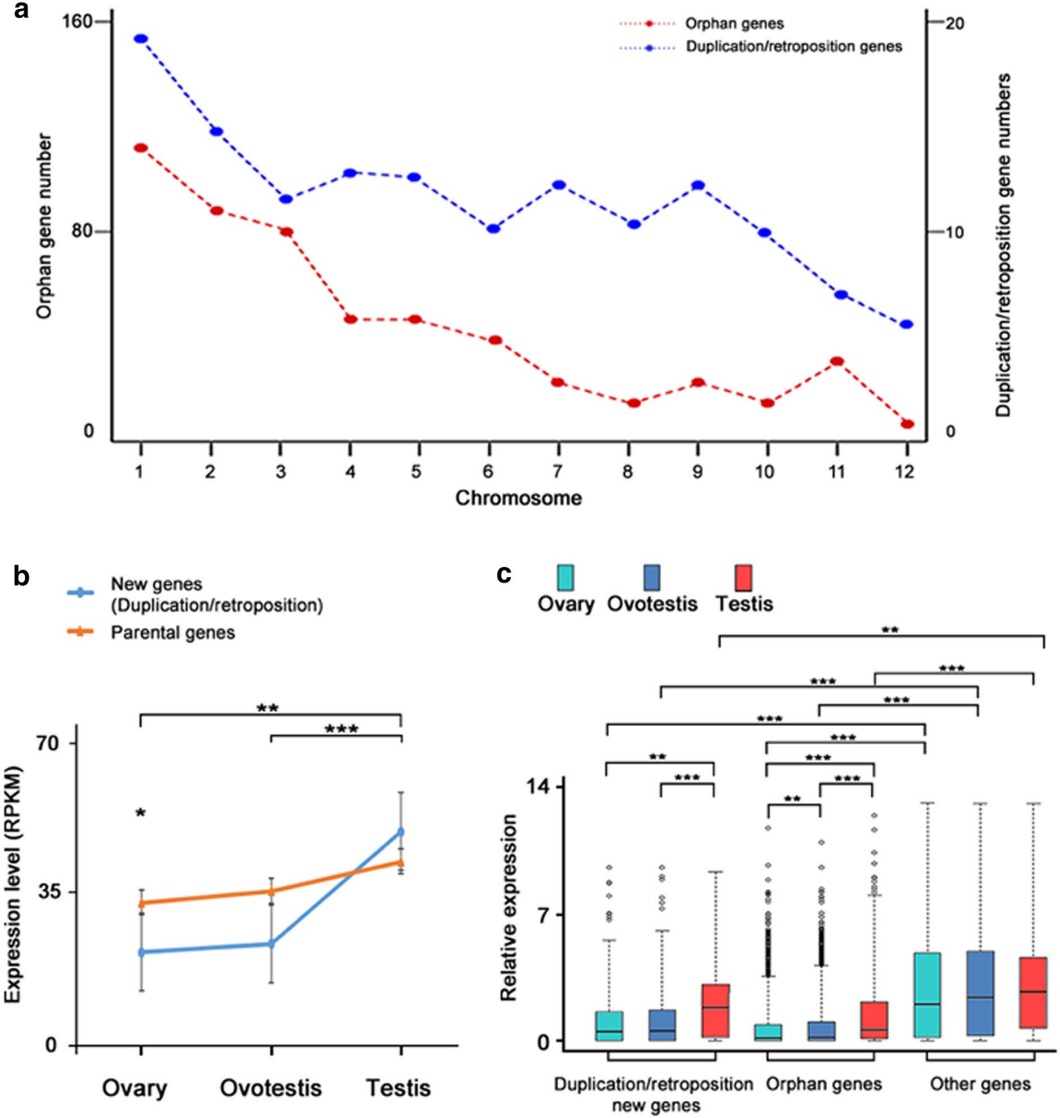
New gene distribution on the chromosomes and upregulation of expression in testis. **a** Distribution of duplication/retroposition genes and orphan genes on chromosomes in *Monopterus albus.***b** Line plots showing average expression levels (RPKM) of duplication/retroposition genes and their parental genes during gonad differentiation. *P* values in the Mann–Whitney U test were calculated for expression level comparisons of new genes among different tissues, and between new genes and parental genes within a tissue. **c** Box plots showing the expression levels (RPKM) of all new genes and orphan genes, in comparison with all other genes during gonad differentiation (Mann–Whitney U test, *p < 0.05, **p < 0.01; ***p < 0.001)

To test whether specific chromosomes are associated with certain detectable patterns of new gene distribution, we mapped these genes to the chromosomes (Additional file 1: Table S3). We found that there was a significantly biased distribution of these new genes, mainly on chromosomes 1, 2, 3 and 4 (χ^2^ test, p < 0.01), whereas a biased distribution of orphan genes was also observed on all 12 chromosomes (χ^2^ test, *p *< 0.05) (Fig. 5a).

We examined the expression levels of new genes, parental genes and orphan genes using the RNA-seq data (Gene Expression Omnibus GSE43649) [28]. A comparison of expression levels among the three stages of gonad differentiation revealed an interesting pattern. In general, we found that the expression levels of new genes and orphan genes were lower than those in the overall distribution of all other genes in gonads, and the expression distributions of new genes showed significantly higher levels for the testis than for the ovary or the ovotestis (*p *< 0.01) (Fig. 5b, c). A significant difference was found in orphan genes, which showed an even stronger trend similar to that found in the new genes. The average expression in the testis was higher than that in the ovary (*p *< 0.001), whereas that in the ovary was significantly different from that in the ovotestis (*p *< 0.01). Overall, newly evolved genes showed significantly elevated expression in the testis. These abundant new genes with high expression in testis emerged in the *Monopterus albus* lineage after split from medaka ~ 70 MYA.

## Discussion

It is becoming increasingly evident that the 3R WGD occurred in the common ancestor of all extant teleosts [4, 29–32]. Afterwards, several teleost linages probably underwent up to six WGD events, particularly in the carp linages [10]. For example, 4R WGD events have been detected in salmonids ~ 80 MYA [48] and the common carp ~ 8.2 MYA [49]. These WGD events could have shaped the history of evolutionary lineages of teleosts, because they create genetic diversity and innovation for adaptive radiation. These WGDs and subsequent diploidization events have tremendous impacts on speciation. Taking advantage of comparative genomics and the genome structure of *Monopterus albus*, here we describe the post-WGD genomic events, evolutionary mechanisms through the whole-genome-wide chromosome fission/fusions, and new gene generations in the *Monopterus albus* lineage. We also reveal evolutionary trajectories of conserved blocks related to sex-determining genes in teleosts.

Genomic variation accumulation can shape the evolutionary direction of lineages under positive selection pressure. During the 2R WGD, the tetrapod (chicken and human) and Holostei (spotted gar) genomes experienced extensive chromosomal fission and block recombination from the ancestral vertebrate genome. The shuffle effect of CSB could be a major cause of block/gene loss after WGD. Moreover, due to accumulation of genomic recombination events, earlier diverged 2R vertebrates retained small CSBs in comparison with 3R teleosts. The same situation applies to 3R teleosts. Zebrafish diverged from other fishes ~ 160 MYA and had smaller CSBs than other teleosts with a short history (< 90 MYA). The variation accumulation is probably different from the mutation accumulation through Muller’s Ratchet observed in asexual lineages [50], as the latter effect often contributes to the extinction of lineages. Instead, the variation accumulation of post-WGD reflects sustaining evolution. In fact, the accumulation of genetic mutations in human adult stem cells was observed in a tissue-specific manner in common mutational processes during life [51], which supports that variation accumulation shapes the direction of lineages during evolution in vertebrates.

The whole-genome-wide chromosome fusion is of particular interest in evolution. The WGCF occurred in the *Monopterus albus* lineage after the 3R WGD. The *Monopterus albus* is distributed worldwide in tropical and subtropical rivers and can naturally change its sex from female to male during its life [7, 8]. The *Monopterus albus* can breathe air, and it is capable of surviving for long periods without water. Its physiological features, including its amphibious abilities, make the species a successful invader around the globe. The WGCF contributes to lower chromosome number among other fish species and speciation through all chromosome fusion. Moreover, a recent finding showed that chromosome fusion events involved another 5 chromosomes that most likely occurred in this species recently, leading to a reduction in chromosome number from 2n = 24 to 2n = 18 [52]. These two types of genetically distinct groups live together in central Thailand. Therefore, sustaining evolution through chromosome fusion events still takes place in the *Monopterus albus* lineage. Intriguingly, chromosomal fission and fusion events were observed in the astronaut after NASA’s 1-year spaceflight [53]. The astronaut postflight was accompanied by cognitive decline, which is often observed in many chromosome syndromes in humans [54]. Chromosome fission/fusion events are probably a tremendous risk of species variation and new speciation.

Evolution of sex determination has been a topic of great interest since Darwin’s time. Our genomic and evolutionary analysis of post-WGD reveal an evolutionary trajectory of sex determination in fish and highlight a role for the teleost Fs chromosomes in female sex determination. Although sex determination systems are variable in vertebrates, there are common genetic components, from genes to chromosomes, shared by the varied systems, owing to the convergent evolutionary pressure. This could explain the inexorable evolutionary processes and underlying mechanisms from labile to entrenched sex chromosomes. In the present study, we trace the evolutionary history of the regions syntenic to the avian Z chromosome from ancestral chromosome E, to the mammalian X from ancestral chromosome G, and to the teleost Fs from ancestral chromosome F in vertebrates. These chromosomes have evolved from different ancestral chromosomes, indicating independent origins from the 2R ancestor (Fig. 3h).

The syntenic *Sox3* block from ancestral chromosome G is conserved, and *Sox3* evolved into the male-determining gene *Sry* on the Y chromosome only in mammals [45, 55], while *Sox3* is essential for ovary development in zebrafish [56]. The syntenic *Dmrt1* block retains from ancestral chromosome E in near all vertebrates, and *Dmrt1* is a major factor for male development in mammals [57], birds [41], reptiles [58], amphibians [59] and fishes [60–62], regardless of its location on the Z/X/Y or autosomes. The Y-linked *dmy* originated from a duplicate copy of autosomal *dmrt1* and can determine male sex in medaka [43, 44]. Over 300 million years, the common ancestral chromosome F evolved a set of descendant chromosomes among the teleost lineages, for example, chromosomes 16 in zebrafish, 8 in *Tetraodon*, 20 in stickleback, 16 in medaka, and 4 in *Monopterus albus*, probably including other teleost fishes, on which *Rspo1*, a pivotal factor for ovary determination [63, 64], has retained. Intriguingly, they inherited historically key components of the Rspo1/Wnt signaling pathway from ancestor chromosome F, including Rspo1, Wnt4b, Ctnnb1 and Gsk3a/b. The pathway is required for ovary development in vertebrates [46, 47]. Consistent with this, breeding experiments showed a female determination signal on chromosome 16 in zebrafish [4]. Together, these data suggest that Rspo1/Wnt signaling components in the common chromosome F are important for female sex determination in the teleost linages.

Molecular mechanisms that generate new genes mainly include DNA-based duplication, retroposition, and de novo origination. Orphan genes have no homologues in closely related lineages. New genes could contribute to the rapid evolution of the genome under positive selection, which favors the success of genetic innovation for adaptive radiation after the 3R WGD. Nevertheless, these candidate new genes identified in *Monopterus albus* remain to be functionally confirmed. Candidate orphan genes should be further determined when more genomes have been sequenced in the related species in the *Monopterus albus* lineage. Genetic studies have shown that the evolution of sex and sex chromosomes impacts the fixation of new genes and their distribution between sex chromosomes and autosomes [65, 66]. Sex-dependent selection has been shown to drive expression divergence between species [67], differentiation of expression level between the X chromosome and autosomes [68] and new gene evolution [40]. The fixation force of new genes was tested as positive selection in the early stages of new genes in *Drosophila* [69] and further confirmed in individual case analyses [70, 71]. The male expression of new genes may be interpreted in terms of male-driven sexual selection [66, 72, 73]. *Monopterus albus* showed a remarkable consistency with the prediction of the Darwin-Bateman paradigm and observations in mammals and insects that males evolve rapidly by recruiting abundant new genes that show biased expression in the testis [40].

## Conclusions

We describe phylogenomic events in teleost fishes including *Monopterus albus* and its relatives after whole-genome duplications, reveal evolutionary mechanisms through the whole-genome-wide chromosome fission/fusions, new gene generations in *Monopterus albus*, and show evolutionary trajectory of conserved blocks related to sex-determining genes, the chromosome Fs for female sex determination in teleosts in particular. These data pave the way for a better understanding of genomic evolution in extant teleosts.

## Supplementary information

**Additional file 1.** Additional figures and tables.

## Data Availability

The Monopterus *albus* whole-genome data were obtained from GenBank under the accession AONE00000000. Raw transcriptome data were obtained from Gene Expression Omnibus as GSE43649.

## Abbreviations

*alkbh6*: alkB homolog 6
*amhr2*: anti-Mullerian hormone type 2 receptor
*AR*: Androgen receptor
CDS: Coding sequence
CSB: Conserved syntenic blocks
*Ctnnb1*: Catenin beta 1
*cyp19a1a*: Cytochrome P450, family 19, subfamily A, polypeptide 1a
DMRT1: Doublesex and mab-3 related transcription factor 1
*dmy*: Doublesex- and mab-3-related transcription factor 1Y-like
Fs: Female sex-determinant F
GO: Gene ontology
*Gsk3a/b*: Glycogen synthase kinase 3 alpha/beta
LGR4/5/6: Leucine-rich repeat-containing G protein-coupled receptor 4/5/6
MYA: Millions of years ago
Rnf43: Ring finger protein 43
RSPO1: R-spondin 1
RPKM: Reads per kb per million reads
*rsu1*: Ras suppressor protein 1
SOX: SRY-box transcription factor
SRY: Sex determining region Y
WGCF: Whole-genome-wide chromosome fusion
WGD: Whole-genome duplication
*Wnt4b*: Wingless-type MMTV integration site family, member 4b
Znrf3: Zinc and ring finger 3
