# Supplementary material for: Whole genome-wide chromosome fusion and new gene birth in the Monopterus albus genome
Source: Cell Biosci. 2020 May 20;10:67. doi: 10.1186/s13578-020-00432-0 (PMC7240998; doi:10.1186/s13578-020-00432-0)
Supplement: Supplementary file 1 — Additional file 1. Additional figures and tables. [file 13578_2020_432_MOESM1_ESM.docx]

**Additional file 1:** Additional figures and tables


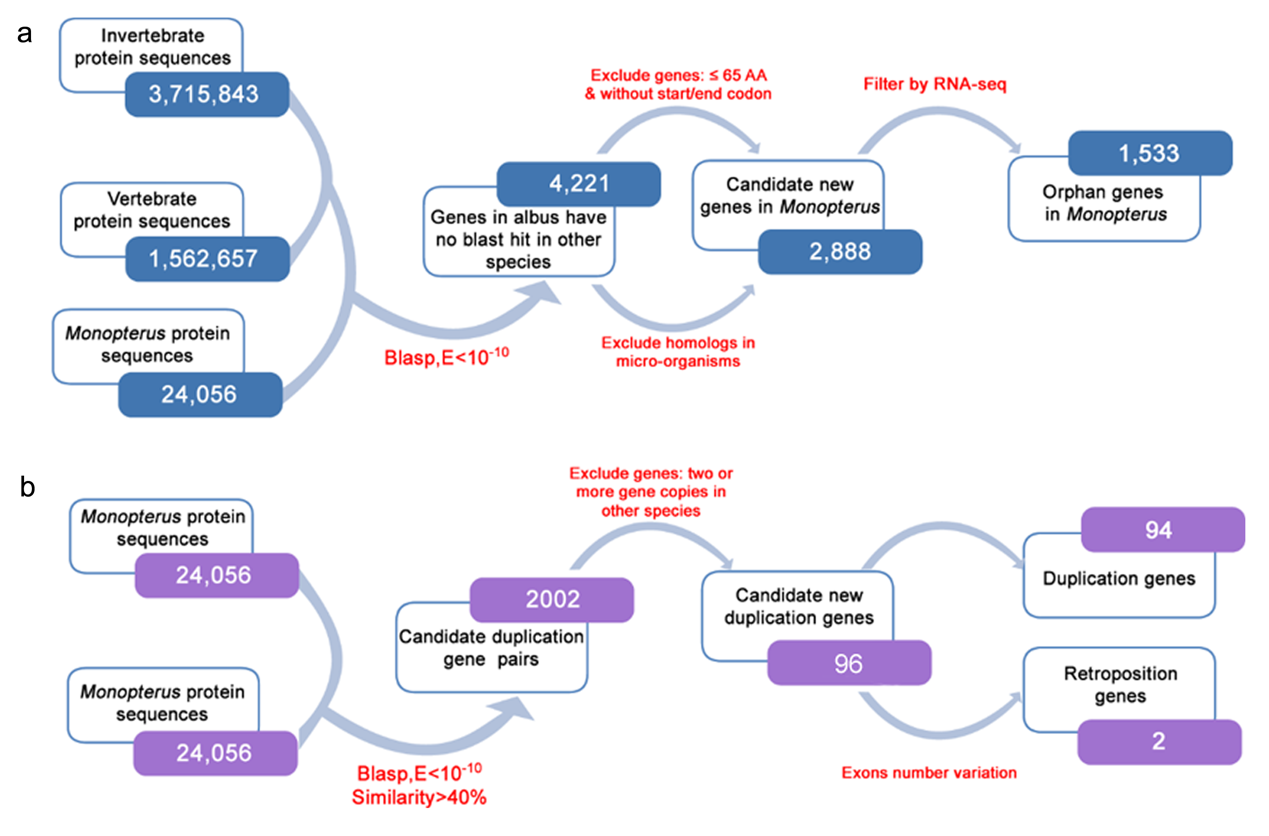
**
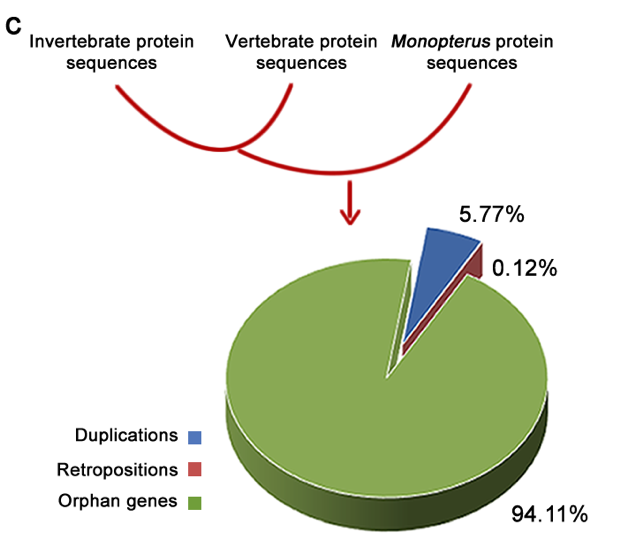
**

**Additional file 1:** Fig. S1 Identification of the new genes.

**a** Pipeline for identification of orphan genes in *Monopterus albus*. 24,056 protein sequences are searched against vertebrate protein sequences (1,562,657) and invertebrate protein sequences (3,715,843) by Blastp. 4,221 genes with no homolog sequences were identified. After excluding the genes of either too short or without start and stop codons, 2,888 genes are candidate orphan genes in *Monopterus albus*. RNA-seq datasets were used to confirm that 1,533 genes are new protein-coding orphan genes. b Pipeline for identification of duplication and retroposition new genes in *Monopterus albus*. 24,056 protein sequences in *Monopterus albus* are self-searched by Blastp. 2002 candidate pairs were identified. These genes are compared with coding genes of other fish species (Tilapia, Medaka, Stickleback, *Tetraodon* and Zebrafish), which makes sure that 96 new genes. By analysis of difference of exon numbers of these genes, we get 94 duplication genes and 2 retroposition genes. c Proportion of new genes and orphan genes. The *Monopterus* *albus* protein sequences were searched against vertebrate protein sequences and invertebrate protein sequences to identify new genes in the *Monopterus* *albus* genome. Generation of new genes occurred after split from medaka approximately ~70.3 MYA. The pie chart shows the percentage of duplication, retroposition and orphan genes in the *Monopterus* *albus* genome.


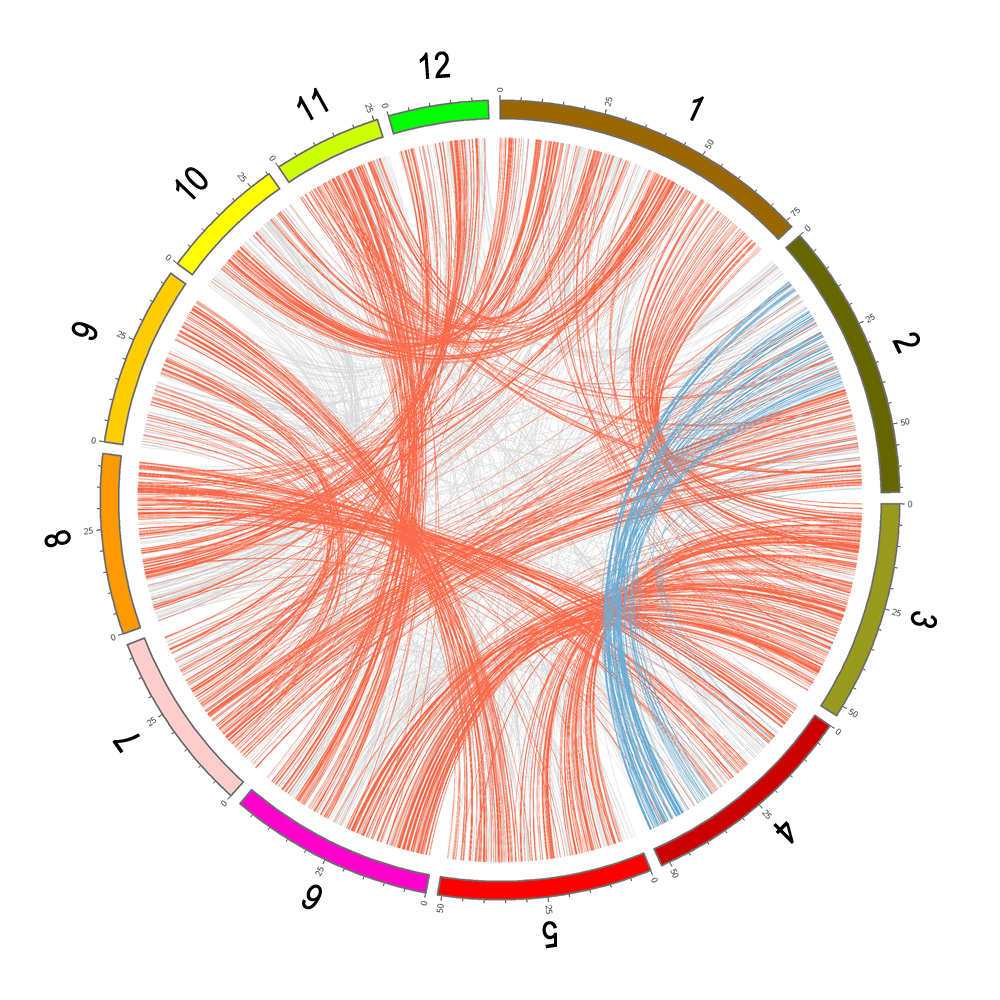


**Additional file 1:** Fig. S2 Circos map showing the gene duplications among chromosomes in *Monopterus albus.*

Outside bars in specific color represent the 12 chromosomes. Scales on the bar describe the relative length of each chromosome in Mb. The colored inner links represent gene pairs on different chromosomes.

**
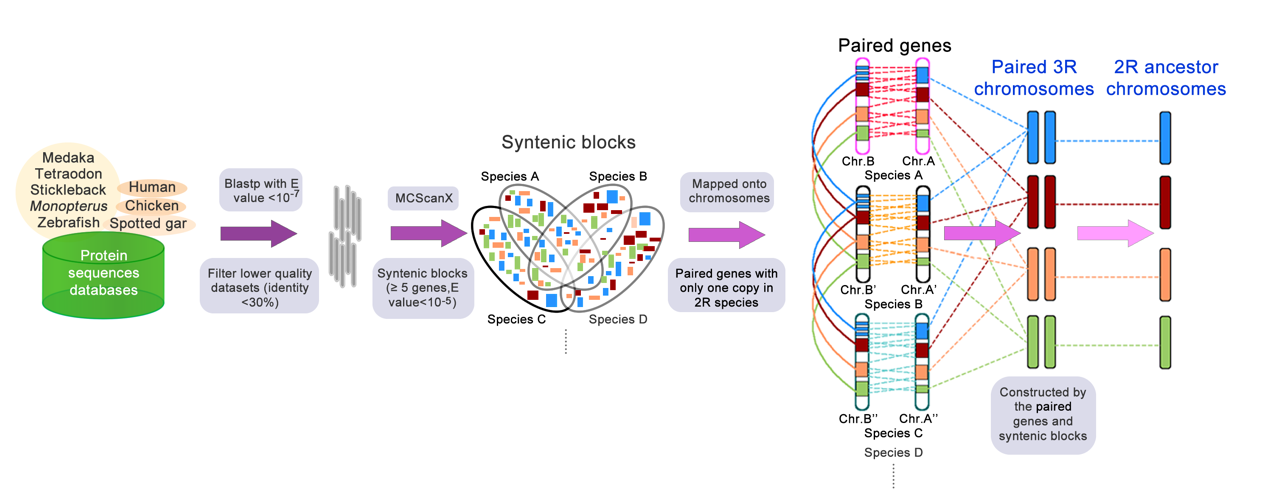
**

**Additional file 1:** Fig. S3 Pipeline for deduction of ancestor chromosomes.

All the protein sequences were aligned to the protein databases of *Monopterus albus* and other species using Blastp with *E* value < 10^-7^, and lower quality datasets (identity <30%) were filtered. MCScanX was used to find conserved syntenic blocks (≥5 genes, E value <10^-5^) between species. The most recent common ancestor chromosomes were deduced, based on the all orthologous syntenic blocks of duplicated gene pairs among species. Conserved blocks in different colors originated from the corresponding ancestor chromosomes.

**
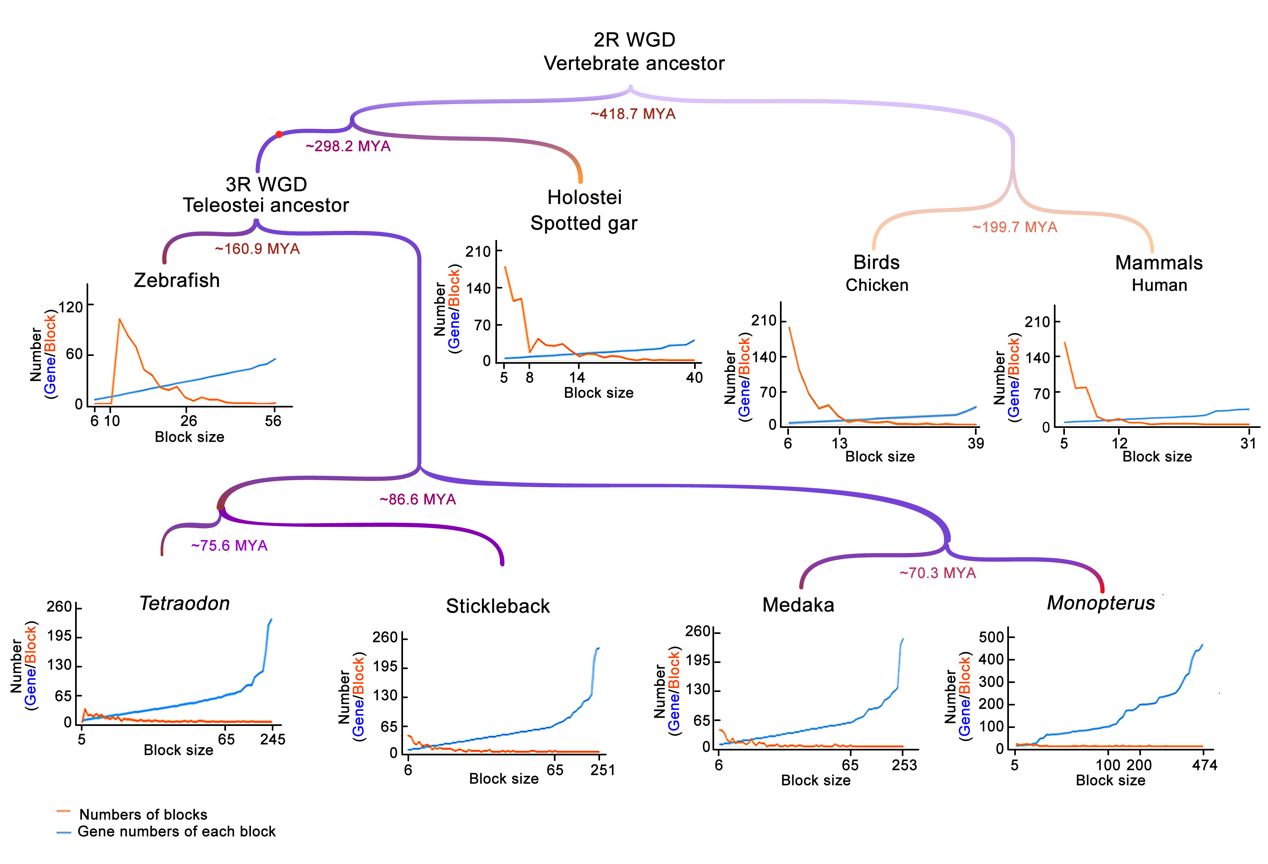
**

**Additional file 1:** Fig. S4 Number and size of conserved syntenic blocks.

The figure depicts the number and size of conserved syntenic blocks of chromosomes in representative species from 2R to 3R: teleosts (*Monopterus albus*, medaka, stickleback, Tetraodon and zebrafish), Holostei (spotted gar), birds (chicken) and mammals (human). The number of blocks is shown in orange, and the gene number of each block is shown in blue. Furthermore. Syntenic blocks with ≥5 genes were included. Average divergence times are shown on the branch nodes.


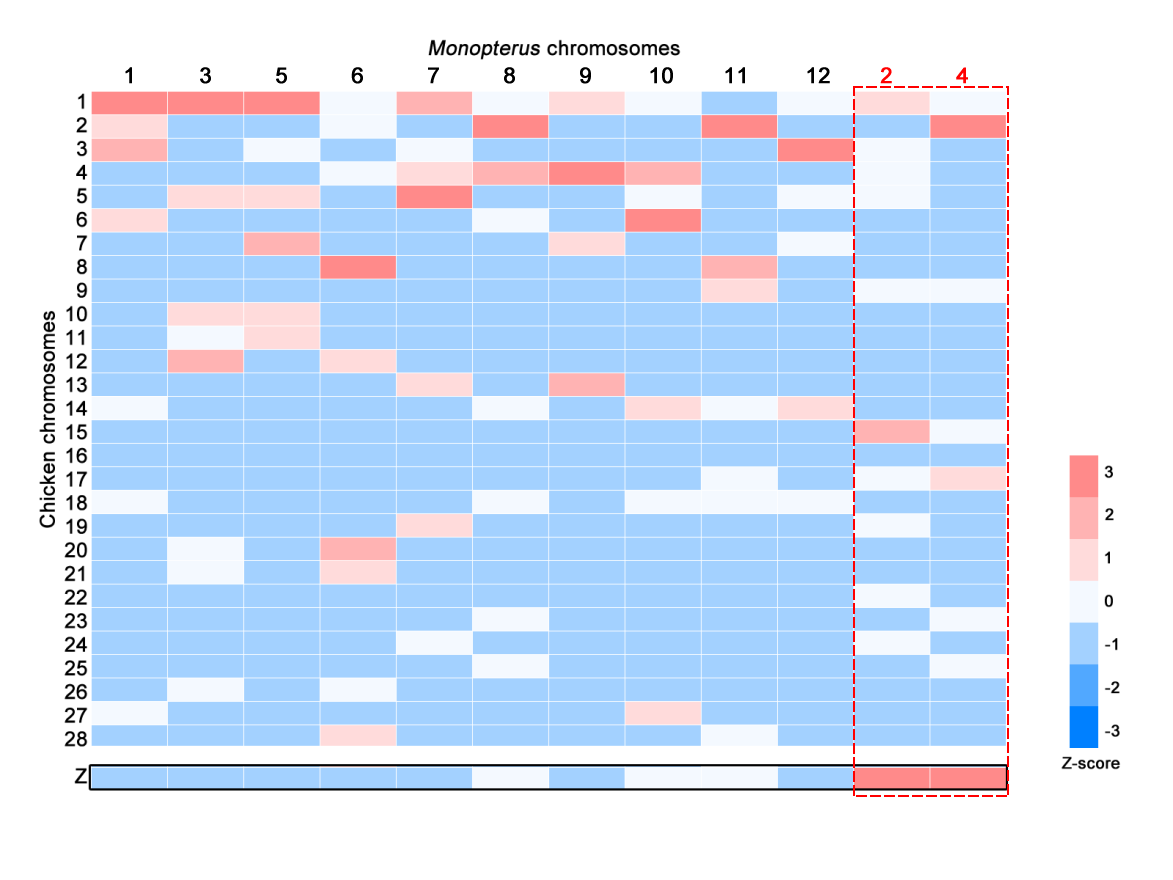


**Additional file 1:** Fig. S5 Chromosome distributions of ortholog genes between chicken and *Monopterus albus*.

Gene numbers between chicken (vertical) and *Monopterus* *albus* (horizontal) are scaled into Z scores with hierarchical clustering. Color key for Z scores is shown on the right side.

**
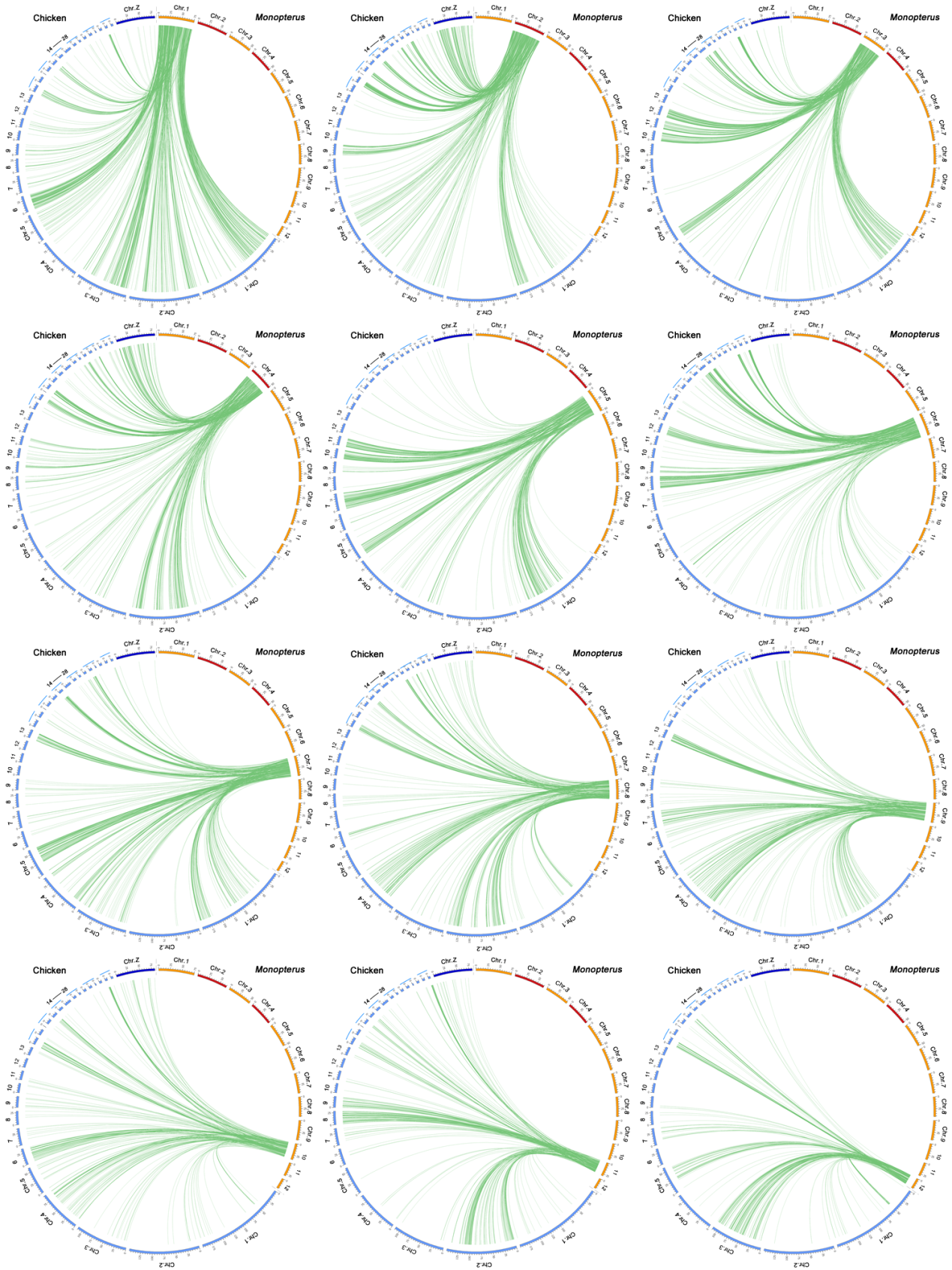
**

**Additional file 1:** Fig. S6 Circos maps show homologous genes in each chromosome of *Monopterus* *albus* with those in chicken chromosomes.

Outside bars in specific color represent the chromosomes. Scales on the bar describe the relative length of each chromosome in Mb. The colored inner links represent gene pairs on chromosomes between chicken and *Monopterus albus*.


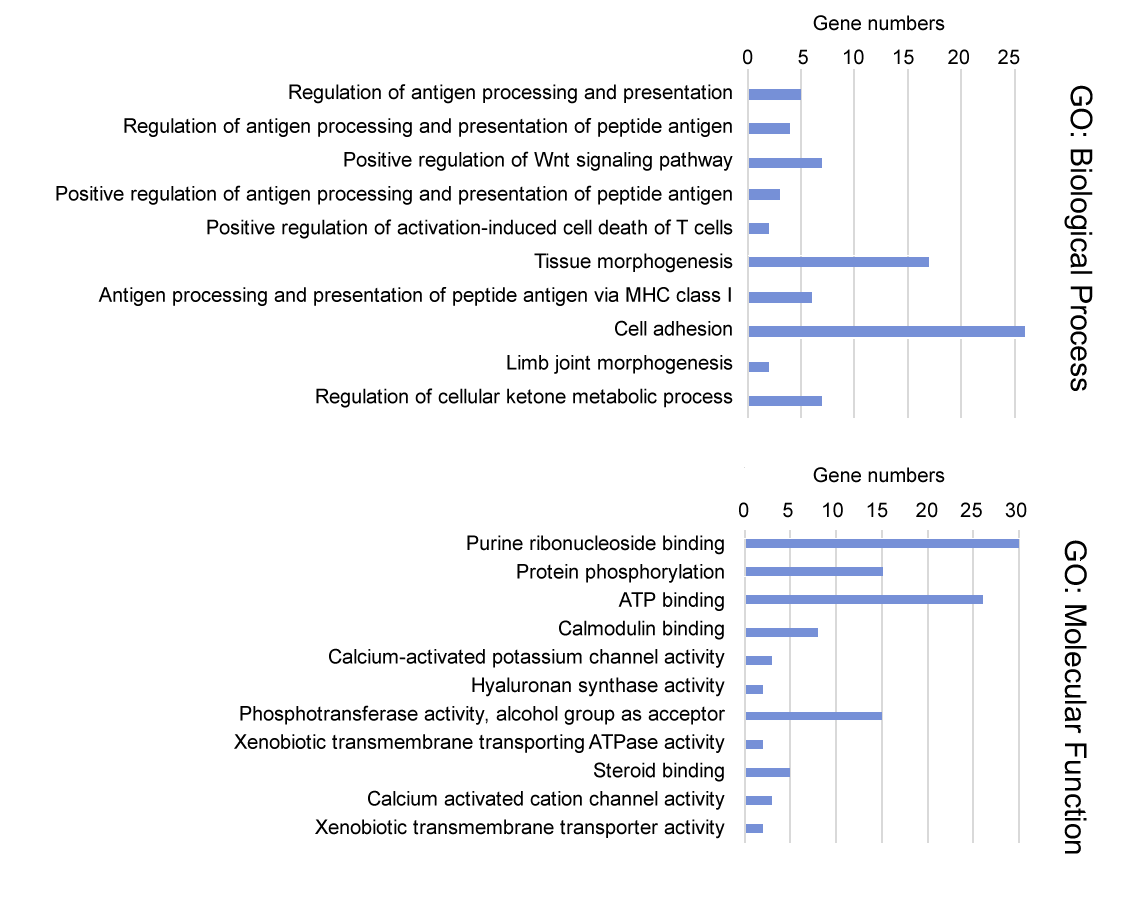


**Additional file 1:** Fig. S7 Gene ontology (GO) analysis of genes on chromosome Fs shared among teleosts.

We used online tool (toppgene.cchmc.org/) to group the GO terms using default parameters.

The catalogs of biological process (FDR: 7.999E-4~2.923E-2) and molecular function (FDR:

4.732E-3~3.833E-2) were listed. Blue bar indicated the number of genes in a category.

**­­­­­**


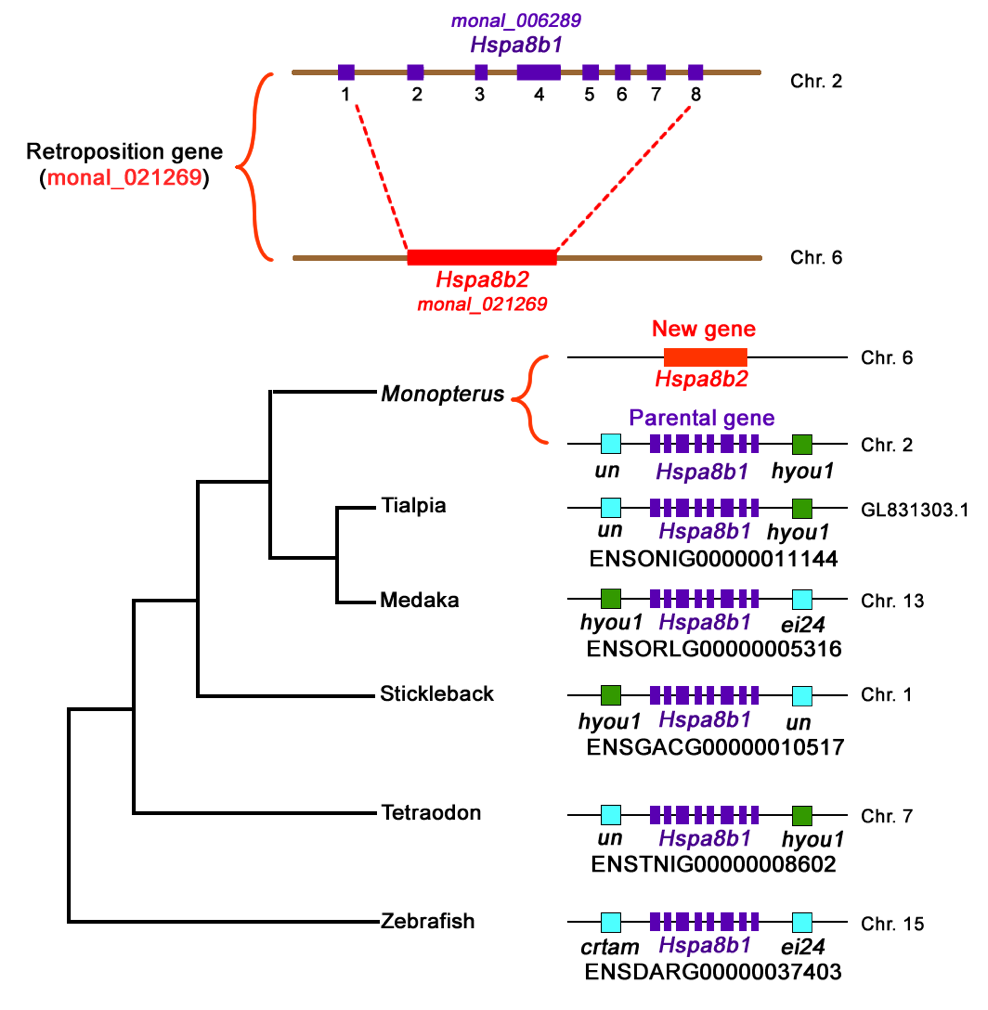


**Additional file 1:** Fig. S8 An example of retroposition gene, *monal_021269.*

The new gene (*monal_021269*) is an RNA-based duplication in which a transcribed RNA is reverse transcribed from the gene *hspa8b1* (*monal_006289­­­*) and retroposed into a new position in the genome. Multiple exons (8 exons) are integrated into one.


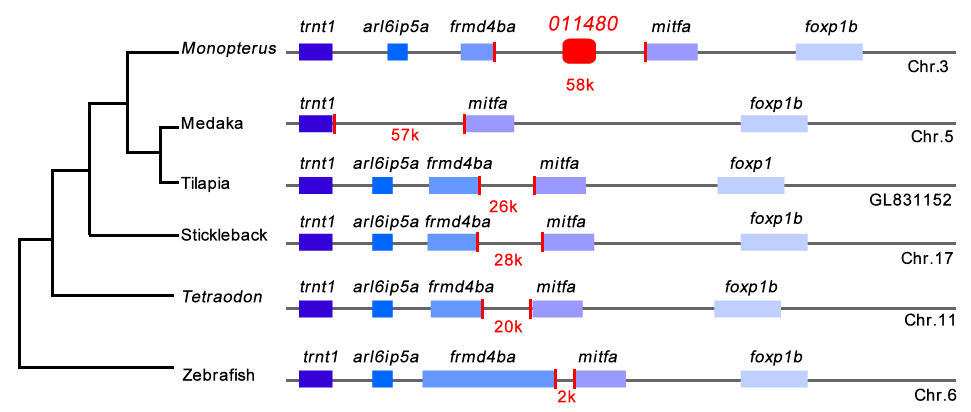


**Additional file 1:** Fig. S9 Characterization of orphan gene, *monal_011480.*

Orphan gene (*monal_011480*) in the genome of *Monopterus albus* has not any homologous genomic region in other species.

**Additional file 1:** Table S1 Chromosome distributions of chicken and *Monopterus albus* orthologous genes

|  |  | *Monopterus albus* chromosomes | | | | | | | | | | | |
| --- | --- | --- | --- | --- | --- | --- | --- | --- | --- | --- | --- | --- | --- |
|  |  | 1 | 2 | 3 | 4 | 5 | 6 | 7 | 8 | 9 | 10 | 11 | 12 |
| Chicken chromosomes | Chr1 | 287 | 105 | 177 | 38 | 201 | 43 | 139 | 36 | 76 | 22 | 13 | 48 |
|  | Chr2 | 155 | 2 | 12 | 200 | 5 | 31 | 3 | 164 | 6 | 8 | 147 | 4 |
|  | Chr3 | 214 | 45 | 11 | 24 | 37 | 5 | 62 | 11 | 17 | 13 | 5 | 191 |
|  | Chr4 | 6 | 42 | 16 | 28 | 9 | 32 | 76 | 134 | 196 | 91 | 7 | 3 |
|  | Chr5 | 36 | 44 | 92 | 5 | 90 | 2 | 193 | 8 | 17 | 17 | 1 | 49 |
|  | Chr6 | 181 | 8 | 1 | 4 | 10 | 1 | 3 | 23 | 10 | 98 | 3 | 0 |
|  | Chr7 | 9 | 3 | 1 | 2 | 168 | 6 | 7 | 1 | 76 | 6 | 11 | 19 |
|  | Chr8 | 13 | 1 | 0 | 24 | 1 | 144 | 4 | 7 | 4 | 3 | 105 | 3 |
|  | Chr9 | 15 | 84 | 0 | 30 | 10 | 23 | 7 | 1 | 6 | 8 | 52 | 4 |
|  | Chr10 | 1 | 0 | 101 | 8 | 131 | 2 | 0 | 2 | 0 | 3 | 1 | 0 |
|  | Chr11 | 14 | 2 | 63 | 17 | 108 | 2 | 0 | 1 | 0 | 4 | 3 | 0 |
|  | Chr12 | 2 | 0 | 131 | 0 | 1 | 73 | 0 | 0 | 0 | 0 | 0 | 0 |
|  | Chr13 | 0 | 2 | 1 | 0 | 0 | 0 | 82 | 0 | 109 | 3 | 3 | 0 |
|  | Chr14 | 51 | 1 | 0 | 3 | 2 | 8 | 0 | 53 | 0 | 65 | 37 | 56 |
|  | Chr15 | 1 | 168 | 9 | 46 | 0 | 2 | 5 | 2 | 2 | 2 | 2 | 1 |
|  | Chr16 | 1 | 0 | 0 | 4 | 0 | 0 | 1 | 1 | 1 | 0 | 2 | 0 |
|  | Chr17 | 3 | 68 | 3 | 100 | 2 | 1 | 1 | 4 | 0 | 2 | 20 | 2 |
|  | Chr18 | 40 | 0 | 1 | 0 | 0 | 3 | 0 | 42 | 0 | 41 | 19 | 33 |
|  | Chr19 | 2 | 79 | 1 | 4 | 2 | 5 | 90 | 1 | 2 | 2 | 2 | 2 |
|  | Chr20 | 1 | 2 | 73 | 2 | 0 | 107 | 0 | 1 | 0 | 1 | 3 | 0 |
|  | Chr21 | 0 | 4 | 51 | 1 | 0 | 93 | 2 | 0 | 0 | 0 | 1 | 0 |
|  | Chr22 | 1 | 53 | 4 | 12 | 1 | 1 | 1 | 0 | 1 | 0 | 0 | 0 |
|  | Chr23 | 4 | 17 | 0 | 44 | 0 | 0 | 17 | 49 | 0 | 0 | 0 | 0 |
|  | Chr24 | 1 | 64 | 1 | 4 | 0 | 0 | 40 | 3 | 1 | 1 | 0 | 0 |
|  | Chr25 | 1 | 1 | 0 | 43 | 1 | 0 | 2 | 30 | 1 | 2 | 4 | 0 |
|  | Chr26 | 0 | 1 | 72 | 0 | 0 | 64 | 0 | 3 | 0 | 0 | 0 | 0 |
|  | Chr27 | 55 | 5 | 1 | 5 | 1 | 3 | 0 | 13 | 2 | 48 | 3 | 2 |
|  | Chr28 | 1 | 1 | 0 | 4 | 1 | 85 | 5 | 3 | 8 | 1 | 37 | 2 |
|  | ChrW | 0 | 0 | 0 | 0 | 0 | 0 | 0 | 0 | 0 | 0 | 0 | 0 |
|  | ChrZ | 7 | 216 | 27 | 178 | 15 | 21 | 4 | 27 | 18 | 24 | 35 | 0 |

**Additional file 1:** Table S2 Identification of duplication/Retroposition new genes

| Similarity | Candidate No. | Confirmed No. |
| --- | --- | --- |
| >40% | 2002 | 96 |
| >45% | 1276 | 88 |
| >60% | 513 | 55 |
| >80% | 203 | 26 |
| >90% | 95 | 17 |

Similarity: new genes via parental genes.

*The results of Similarity >40% are confirmed as duplication new genes.

**Additional file 1:** Table S3 New genes numbers on chromosomes

| Chr. | New gene No. | %. |
| --- | --- | --- |
| 1 | 13 | 13.54% |
| 2 | 11 | 11.46% |
| 3 | 10 | 10.42% |
| 4 | 6 | 6.25% |
| 5 | 6 | 6.25% |
| 6 | 5 | 5.21% |
| 7 | 3 | 3.23% |
| 8 | 2 | 2.08% |
| 9 | 3 | 3.23% |
| 10 | 2 | 2.08% |
| 11 | 4 | 4.17% |
| 12 | 1 | 1.04% |
